# Job Satisfaction Among Pharmacists Graduating from a University in Northern Sweden: A Comparative Analysis

**DOI:** 10.3390/pharmacy13060171

**Published:** 2025-11-22

**Authors:** Maria Gustafsson, Helena Norberg, Sofia Mattsson

**Affiliations:** Department of Medical and Translational Biology, Umeå University, SE-901 87 Umeå, Sweden; maria.gustafsson@umu.se (M.G.); helena.norberg@umu.se (H.N.)

**Keywords:** pharmacist, pharmacy graduate, job satisfaction, continuing professional development

## Abstract

Job satisfaction plays a critical role in shaping professional outcomes, as it has been positively associated with enhanced performance and greater motivation. Conversely, insufficient job satisfaction may contribute to higher rates of staff turnover, professional burnout, and intentions to leave the profession. The objective was to investigate job satisfaction among pharmacists educated at Umeå University in Sweden over time and to explore factors affecting job satisfaction. A survey was distributed to pharmacy graduates who had completed web-based pharmacy programs at Umeå University between 2019 and 2023. Questions regarding job satisfaction and factors related to it were included. The response rate was 38%. The results were compared with results from a previous investigation (graduation years 2015–2018) to enable comparisons over time. Compared to findings from the previous survey, job satisfaction was lower in the present study (76.4% vs. 91.4%, *p* = 0.004). Both greater opportunities for continuing professional development (CPD) and the perception that the knowledge and skills gained during education are beneficial in the current job were associated with high job satisfaction (OR: 5.360; 95% CI: 1.896–15.156 and OR: 3.983; 95% CI: 1.255–12.642, respectively). Understanding factors contributing to job satisfaction can help employers improve retention and work environment.

## 1. Introduction

Pharmacists are considered an integrated part of the healthcare system and work in community and hospital pharmacies and in clinical settings. Furthermore, pharmacists are also employed in the pharmaceutical industry, academia and authorities such as medical agencies. In recent years, healthcare systems have evolved with an increased focus on optimizing quality of care, ensuring patient safety and strengthening the provision of patient-centered care [1]. The development of clinical pharmacy and pharmaceutical care has contributed to the advancement of the pharmaceutical profession. This development has led to a transformation in the role of pharmacists in Sweden, shifting from a primary focus on drug dispensing to active clinical involvement within multidisciplinary ward teams in both hospital and primary care settings. A reregulation of the Swedish pharmacies took place in 2009, when the state-owned pharmacy monopoly was replaced by an open market allowing private pharmacy chains to operate [2,3]. By the end of 2022, the number of community pharmacies in Sweden had reached 1407, representing an increase of 480 pharmacies (an increase of over 50%), compared to 2009, prior to the reregulation of the pharmacy market [4]. From 2009 to 2022, pharmacy density rose from 10 to 13.4 per 100,000 inhabitants [2]. This transition affected both community and hospital pharmacies, with the potential to affect job satisfaction among pharmacists. Due to the expansion of community pharmacies, the demand for licensed pharmacists is high, and there is a shortage of pharmacists in Sweden, especially in rural and remote areas.

Job satisfaction among pharmacists has been investigated previously, and the level of job satisfaction has varied. A study from Virginia, USA, showed that 86% of the pharmacists were very or somewhat satisfied with their job [5], whereas a study from Northern Ireland showed that just above 50% of pharmacists working in hospital and community pharmacies were satisfied with their job most of the time [6]. A study from Malaysia found that community pharmacists expressed a high level of satisfaction with their work, with 77% reporting job satisfaction [7], while another study from Malaysia focusing on pharmacists in the public sector reported a lower proportion of satisfied respondents (52%) [8]. Two studies from Sweden showed that most of the participating pharmacists (91%) reported being satisfied with their job most or all of the time [9,10].

Job satisfaction is important since it may affect performance, productivity and motivation in a positive way, whereas job dissatisfaction may affect patient safety and care in a negative way and increase job turnover, burnout and the intention to leave the profession [11,12,13,14,15,16,17,18]. Several factors affecting job satisfaction have been identified in previous studies, both on an organizational and personal level. For example, work environment, salary, benefits, career development, continuous professional development, age, work experience, and level of education may have an impact on job satisfaction [19]. A recent review discussed key factors influencing job satisfaction among pharmacists, and these factors included work conditions and roles, stress and workload, burnout, professional development, earnings and benefits, and leadership support [20].

There are reports of shortcomings in work conditions, especially at community pharmacies in Sweden. A limited interest among pharmacists in pursuing careers within Swedish community pharmacies has resulted in staffing shortages and unfilled positions. Consequently, pharmacy chains are often unable to expand as planned, and in some cases, existing pharmacies, particularly in rural and remote areas, have been forced to close due to a lack of qualified personnel [4,21]. On an international level, research indicates that recruitment and retention of pharmacists in rural regions is further challenged by professional isolation, limited peer support, and difficulties in maintaining a healthy work–life balance, often due to challenges in taking leave [22]. Furthermore, occupational health and safety surveys conducted by trade unions in Sweden have highlighted deficiencies in working conditions, which may contribute to pharmacists’ reluctance to work in community pharmacy settings [4,21]. Although two Swedish studies indicate a decline in job satisfaction among pharmacists [10,23], the overall body of research on this topic remains limited in Sweden. Given recent reports highlighting deficiencies in pharmacists’ working conditions, further analysis is warranted to investigate whether these developments may have an impact on job satisfaction. The objective of the present study was therefore to investigate job satisfaction among pharmacists graduating from Umeå University in Sweden over time and to explore factors affecting job satisfaction.

## 2. Materials and Methods

### 2.1. Setting

Umeå University is one of five public universities in Sweden that offer pharmacy education. Three pharmacy programs are given at Umeå University: a three-year Bachelor of Science in Pharmacy program, a five-year Master of Science in Pharmacy program and a two-year Master of Science in Pharmaceutical Science program. Graduates holding a Bachelor of Science in Pharmacy degree are eligible to apply for the two-year master’s program to obtain a master’s degree. The pharmacy curricula include courses in, for example, chemistry, biomedical science, pharmacology, pharmacotherapy, pharmaceutics, social pharmacy, clinical pharmacy and pharmacy practice. Approximately 400 students, of which about 75 are from Umeå University, graduate annually from pharmacy programs at Swedish universities [24]. There are two different professional degree levels in pharmacy in Sweden; prescriptionists, who hold a bachelor’s degree, and pharmacists, who hold a master’s degree. License to practice as a prescriptionist or pharmacist is issued by the National Board of Health and Welfare in Sweden and there was approximately 11,000 registered prescriptionists and pharmacists in 2022 [25]. The term pharmacist will be used henceforth to describe both professional degrees.

### 2.2. Alumni Survey

A cross-sectional study among graduates from the pharmacy programs at Umeå University in Sweden was performed. All graduates from the three pharmacy programs graduating between 1 January 2019 and 30 September 2023 were invited to participate. The total number of graduates during the selected period was 330. A paper questionnaire was sent to the graduates in October 2023. The university’s administrative record was used to collect current addresses. The university’s administrative record is continuously updated with address information provided by the Swedish Tax Agency, the authority responsible for civil registration of individuals in Sweden. The participants were given the option to send in the filled questionnaire using a postage-paid envelope or to answer the questionnaire using a QR-code and fill out the form digitally. Two reminders were sent by e-mail using the registered e-amil address in the university’s administrative records. Data was collected during October and November 2023. One hundred and twenty-five graduates completed the questionnaire either by post (21 graduates) or digitally (104 graduates), which gave a response rate of 38%.

Alumni investigations have previously been conducted among pharmacy graduates at Umeå University, one among graduates with graduation years 2006–2014 [9] and the other among graduates with graduation years 2015–2018 [10]. The questionnaire used in these previous studies as well as in the present study was first developed at Umeå University based on literature on job satisfaction and other alumni surveys from the university [9]. The questionnaire included questions about the graduates’ current employment and work assignments, their level of job satisfaction, and their education. A five-item validated version of the survey from McCann et al. was used to evaluate job satisfaction [6]. These specific questions on job satisfaction were translated into Swedish and then back translated into English and pilot tested [9]. The same questions were used to evaluate job satisfaction in the previous alumni studies from Umeå University [9,10]. In a previous study, results regarding job satisfaction were compared between graduates with graduation years 2006–2014 and 2015–2018 [10]. In the present study, the results regarding job satisfaction were compared with results from the most recent survey, i.e., pharmacy graduates with graduation years 2015–2018. The methodology used in the present study is identical to that of the previous one, which enables a comparison [10].

### 2.3. Data Analysis

Job satisfaction was evaluated using the question: “All things considered, how often are you satisfied with your job?”. For analysis, responses were dichotomized into two categories; “not satisfied” (including “never or rarely” and “sometimes satisfied”) and “satisfied” (including “satisfied most of the time” and “satisfied all of the time”). The questions “how often do you find the idea of spending the rest of your working life in a job like your current one depressing”, “how often do you leave work with a “bad” feeling”, and “how often do you get so absorbed in your work that you lose track of time” were all dichotomized into “never/sometimes” (including “never or rarely” and “sometimes”) and “most of the time/all of the time” (including “most of the time” and “all of the time”). Opportunities for continuing professional development (CPD) was categorized as “limited” (including “limited” and “very limited”) and “good” (including “satisfactory”, “good”, and “very good”). The question regarding whether “the knowledge and skills gained during your education are beneficial in your current job” was dichotomized based on a 5-point scale: scores of 1–3 were classified as “disagree”, and scores of 4–5 as “agree”. Group differences were analyzed using the chi-squared test.

To find factors independently associated with job satisfaction, first, simple logistic regression analyses were performed including the dependent factor job satisfaction, and one independent factor at a time. The independent factors were continuous factors; age and time since graduation, and categorical factors; gender, employment category, workplace, income, opportunities for CPD, and the perceived relevance of knowledge and skills gained during education to the current role. These factors were selected based on prior research indicating their association with job satisfaction [8,26,27,28,29]. Subsequently, a multiple logistic regression analysis was performed, with the dependent variable job satisfaction and the independent variables that were statistically significant in the simple models, along with age and gender. Results are reported as odds ratios (ORs) with 95% confidence intervals (CIs). A significance level of 0.05 was applied to all statistical tests. All analyses were performed with Statistical Package for the Social Sciences (SPSS) for Windows, version 29 (IBM, Armonk, NY, USA).

## 3. Results

### 3.1. Demographics

Most of the respondents were women, and the average age was 34 years (Table 1). Approximately two-thirds of the graduates participating in the survey were born in Sweden. Most of the participating graduates (67.5%) worked at a community pharmacy. A higher proportion of graduates holding a master’s degree were employed in county council and pharmaceutical industry sectors compared with those holding a bachelor’s degree. Most of the participating graduates held permanent positions and secured their first employment prior to graduation. For comparison, characteristics of participating graduates in the previous survey (graduation years 2015–2018) are included in Table 1.

### 3.2. Job Satisfaction

The results indicated that most participants (76.4%) reported being satisfied with their job, selecting the response options “most of the time” or “all of the time” to the question regarding overall job satisfaction (Table 2). Compared to findings from the previous survey conducted at Umeå University among graduates with graduation years 2015–2018, job satisfaction was significantly lower in the current study (76.4% vs. 91.4%, *p* = 0.004). Regarding the question “ How often do you think the idea of spending the remainder of your working life in a job like your current one is depressing?” no significant difference was found between the groups (21.6% vs. 12.8% chose “most of the time” or “all of the time” to the question, *p* = 0.094). However, a significantly larger proportion of participants in the current study reported that they leave work with a “bad” feeling compared to the previous survey (12.0% vs. 4.26% chose “most of the time” or “all of the time” to the question, *p* = 0.043). Regarding the question “How often do you get so wrapped up (interested) in your work that you lose track of time?” there was no significant difference between the two groups (36% vs. 39.2% chose “most of the time” or “all of the time” to the question, *p* = 0.703). When comparing job satisfaction between graduates with a bachelor’s and those with a master’s degree, no significant differences were observed in the present study (*p* > 0.05).

In response to the question of whether they would choose pharmacy again if they could restart their career, 39.0% of respondents chose “definitely yes” and 29.2% “maybe”. In both the current and previous study, the majority responded either “definitely yes” or “maybe” to the question (68.3% vs. 80.6%, *p* = 0.041); however, a lower proportion indicated that they would choose pharmacy again in this study as opposed to the findings in the study among graduates with graduation years 2015–2018.

The respondents were also asked about opportunities for CPD in their current positions, and the majority perceived these opportunities as high (i.e., satisfactory to very good). In total, 69.7% of respondents in the current study reported that the opportunities for CPD was high, compared to 64.9% in the previous study among graduates with graduation years 2015–2018 (*p* = 0.525). In response to the question of whether the knowledge and skills gained during education are beneficial in my current job, 77.4% of participants answered “completely agree” or “mostly agree,” compared to 82.6% in the previous study among graduates with graduation years 2015–2018 (*p* = 0.349).

To investigate how different factors affect job satisfaction, a regression analysis was performed (Table 3). Working in community pharmacy, greater opportunities for CPD and perceiving that the knowledge and skills gained during education are beneficial in the current job, were associated with higher job satisfaction in the simple regression analysis but no associations between other factors and job satisfaction were seen. Greater opportunities for CPD (OR: 5.360; 95% CI: 1.896–15.156) and the perception that the knowledge and skills gained during education are beneficial in the current job (OR: 3.983; 95% CI: 1.255–12.642), were associated with high job satisfaction in the multiple regression.

## 4. Discussion

This study investigated how job satisfaction among pharmacists educated at Umeå University has changed over time by comparing two groups: those who graduated between 2015 and 2018 and those who graduated between 2019 and 2023. The main finding was that although job satisfaction was relatively high, a notable decrease in job satisfaction has occurred since the last investigation.

The characteristics of the graduates in the present study resembled those previously reported in pharmacy alumni surveys at Umeå University [9,10]. Most graduates were female, consistent with the gender distribution of the pharmacy workforce in Sweden and globally [30,31,32,33]. Furthermore, most graduates were Swedish-born and resided in Sweden. The mean age of the respondents was 34 years (median 31 years), and the mean time since graduation was 1.8 years. In comparison, graduates in Sweden have a median age of 27.1 years (2023/2024) [31]. The slightly higher age of the graduates is likely due to the web-based format of the pharmacy programs at Umeå University, which tends to attract older students [34]. Most of the respondents worked at a community pharmacy. Since the previous survey among pharmacy graduates with graduation years 2015–2018, the proportion working in county councils and the pharmaceutical industry has increased slightly [10]. A higher proportion of graduates with a master’s degree worked in these sectors compared with those holding a bachelor’s degree, which may be expected since the master’s program allows for a more comprehensive education, allowing students to work with more extensive and varied tasks. Although the pharmacists participating in the survey all graduated from the same university in northern Sweden, their current workplaces are distributed across the entire country.

Job satisfaction among the participants was generally high (76.4% of the graduates reported being satisfied with their job “most of the time” or “all the time” to the question about overall job satisfaction), which is in line with or higher than previous research [5,6,7,8,35]. Although a relatively high level of job satisfaction was reported, job satisfaction has significantly decreased since the last investigation at Umeå University, where 91.4% of the graduates (graduation years 2015–2018) reported being satisfied with their job “most of the time” or “all the time”. A significantly higher proportion of graduates responded “most of the time” or “all of the time” to the question “how often do you leave work with a “bad” feeling, a feeling that you are doing something you do not enjoy” in the present study as opposed to the previous study (graduation years 2015–2018). The overall decrease in job satisfaction represented a notable deviation from the trend observed in the previous studies from Umeå University (graduation years 2006–2014 and graduation years 2015–2018), where no difference was observed (91.2% vs. 91.4%, *p* = 0.965) [10]. Another study has also shown that job satisfaction among pharmacists in Sweden has decreased [23]. Further, a study from New Zealand showed that job satisfaction had not improved over the last two decades [36].

A positive relationship between work environment and job satisfaction has been observed in the literature [18,19,20]. In the present study, factors related to the work environment, such as workload, stress, and leadership support, were not explicitly investigated. However, it could be argued that one reason for the observed decrease in job satisfaction may be related to the reported work environment issues in Swedish community pharmacies [21,23,37]. The increased demand for pharmacists due to the increased number of community pharmacies may create an increased workload, which can affect job satisfaction negatively. This may contribute to pharmacists’ reluctance to work in community pharmacy settings, which can further aggravate the current pharmacist shortage in community pharmacies, thus increasing work-related stress even further. Factors such as shortage of staff, high demands, and insufficient support from employers contribute to occupational stress and increased levels of work-related unhealth and decreased job satisfaction [20,38]. Heightened workplace stress, growing administrative demands within community pharmacies, a strained healthcare infrastructure, and an intensifying commercial orientation have been identified as contributing factors that may negatively influence pharmacists’ job satisfaction in community pharmacy settings globally [36,39,40,41].

The decrease in job satisfaction observed in the present study may also explain why there was a significant decrease in the proportion of graduates who would choose pharmacy again if they were to restart their careers. This represented a notable deviation from the trend observed in the previous studies from Umeå University (graduation years 2006–2014 and 2015–2018), where no differences were observed with regard to this question [10]. The decline may reflect an increased awareness of the challenges associated with the profession, including high workload, stress, and limited opportunities for career advancement. Previous research has demonstrated that factors such as work environment and employment conditions play a crucial role in career choice and sustained professional motivation [42]. Moreover, changes in the labor market and expectations regarding the pharmacist’s role may have influenced respondents’ attitudes. In addition, some of the respondents began their professional careers as pharmacists during the COVID-19 pandemic, which may also have influenced the results in the present study due to the unprecedented circumstances arising from the pandemic [43].

Previous studies have found that education level may affect job satisfaction among pharmacists and that people with a higher education level were generally more satisfied with their job [44,45]. However, in the present study there were no differences in job satisfaction between bachelor’s and master’s graduates.

A regression analysis was performed to identify potential factors affecting job satisfaction. In the multiple regression analysis, greater opportunities for CPD and the perception that the knowledge and skills gained during education are beneficial in the current job were significantly associated with higher job satisfaction. None of the other investigated factors (i.e., age, gender, employment category, workplace, time since graduation and income) affected job satisfaction in the present study.

A majority of the respondents in the study were employed in community pharmacies. The simple regression analysis indicated that pharmacists working in community pharmacy reported lower job satisfaction compared to pharmacists employed in other sectors; however, this association was not statistically significant in the multiple regression analysis. The effect of the workplace on job satisfaction has been studied previously. One study showed that pharmacists working in chain community pharmacies reported lower job satisfaction in comparison to other workplaces, including independent community pharmacies [5]. This was explained by increased workload, elevated work-related stress and reduced opportunities for rest and engagement with co-workers due to a shortage of staff in chain community pharmacies. A study from Great Britain similarly found that community pharmacists reported lower job satisfaction compared to their counterparts in other sectors [26]. However, another study found that pharmacists working in chain pharmacies reported higher job satisfaction compared to those employed in independent pharmacies [7]. The impact of different workplaces on job satisfaction seems to differ, although it could be expected that work environments may differ across workplaces, which could potentially affect job satisfaction. The lack of an observed workplace effect on job satisfaction in the present study could be related to a relatively small number of respondents working outside community pharmacy.

The present study did not find any significant associations between job satisfaction and income. This is in line with the previous studies conducted at Umeå University [9,10]. In contrast, having a high income was associated with higher job satisfaction, according to two studies performed among pharmacists in the USA [5,28]. Further, gender and age can affect job satisfaction, and women and older people have generally reported higher job satisfaction than men and younger people [8,9,26,29,46,47]. In addition, a U-shaped relationship between age and job satisfaction has been reported, showing that both younger and older people tend to experience higher job satisfaction compared to those in midlife [29]. However, the present study found no significant associations between job satisfaction and either age or gender. This may be attributable to the sample size and the limited age range of the respondents.

A majority of the respondents perceived that the knowledge and skills gained during their education were relevant to their current professional role, and this was also associated with higher levels of job satisfaction. Additionally, greater opportunities for CPD were associated with higher job satisfaction in the present study, consistent with findings from previous studies at Umeå University [9,10]. In addition, this has also been observed internationally [20,27]. In other words, CPD represents an important factor for employers to consider, as it is strongly associated with job satisfaction.

In the present study, job satisfaction was not associated with time since graduation. This contrasts with a previous study at Umeå University, where job satisfaction among pharmacists appeared to decrease with increasing years in the profession, possibly due to lack of career paths and opportunities [9]. A likely explanation for this difference in results could be that the time span during which participants graduated was narrower in the present study (2019 to 2023 compared to 2006 to 2014 in the previous study). Another study reported that newly graduated pharmacists had a lower job satisfaction than those with more than five years since graduation [23]. This may be due to incongruence between new graduates’ expectations and real-world experience [48,49,50]. Conversely, a meta-analysis found no significant difference in job satisfaction between pharmacists with ten years or less of work experience and those with more extensive experience. In the present study, all participants were relatively recent graduates, making this kind of comparison difficult. To better capture the effect of time since graduation on job satisfaction among pharmacists, a more diverse group in terms of work experience would have to be investigated.

This study has both strengths and limitations. One notable strength of the study is its ability to assess job satisfaction over a relatively long time period, and the use of identical questions regarding job satisfaction across cohorts enabled comparisons over time. However, job satisfaction is inherently subjective and may vary depending on individual preferences and cultural factors, which can influence how job satisfaction is perceived. Because job satisfaction was assessed at only one point in time, this limitation must be taken into account when evaluating the findings. Furthermore, the findings may not be fully generalizable to all pharmacists in Sweden, as the study sample consisted solely of graduates from a single university. All respondents were included in the analysis of job satisfaction, including those who were on parental leave or students. Those who were on parental leave or students likely responded to the job satisfaction questions based on their previous work experiences as pharmacists or their part-time work as pharmacists while pursuing their master’s degree. Since the survey was distributed in October 2023 to those graduating between January 2019 and September 2023, some respondents have limited professional experience, which may influence the reliability of their answers. Finally, the relatively low response rate can affect the representativeness of the results and the generalizability of the findings. The possibility of selection bias cannot be ruled out, as graduates with a more positive attitude towards their profession may have been more inclined to respond to the survey than those with a more negative attitude.

## 5. Conclusions

The most important finding from this study is that job satisfaction has declined among pharmacy graduates. The study nevertheless found that job satisfaction was still relatively high. Opportunities for CPD and the perceived relevance of knowledge and skills gained during education emerged as significant predictors of job satisfaction. No statistically significant effects of gender or age were observed. Furthermore, factors such as income, employment category, workplace, and time since graduation did not demonstrate any notable impact on job satisfaction. The findings of this study provide an opportunity to advance the understanding of pharmacists’ job satisfaction in Sweden and may contribute to elucidating the observed negative trend in job satisfaction. Elevated job satisfaction among pharmacists may contribute to enhanced performance, increased motivation, and greater productivity, while low satisfaction can negatively impact patient care and increase staff turnover. Understanding its determinants is crucial for employers and policymakers aiming to improve retention and work environments and can also guide educational institutions in preparing graduates for professional practice. Future research should focus on investigating job satisfaction in a broader context, thereby providing insights into job satisfaction among pharmacists in Sweden in general. Additionally, work environment factors such as workload, stress and autonomy should be included in the survey to provide a more comprehensive understanding of their impact on job satisfaction among pharmacists in Sweden.

## Figures and Tables

**Table 1:** Characteristics of participating pharmacy graduates (graduation years 2019–2023). Characteristics of participating pharmacy graduates with graduation years 2015–2018 are included for comparison (data from [10]).

Characteristics	All Graduates (Graduation Years 2019–2023) *n* = 125	Bachelor’s Degree (Graduation Years 2019–2023) *n* = 70	Master’s Degree (Graduation Years 2019–2023) *n* = 55	All Graduates (Graduation Years 2015–2018) *n* = 94
*Gender (%)*				
Women	87.9	85.7	90.7	92.6
*Age (years)*				
Mean (standard deviation)	34.0 (8.8)	33.2 (8.9)	35.1 (8.7)	35.0 (8.6)
Range	21–58	21–57	24–58	
*Country of birth (%)*				
Sweden	65.0	69.1	59.4	77.2
Outside Sweden	35.0	30.9	40.6	22.8
*Country of living (%)*				
Sweden	99.2	100.0	98.8	98.9
Outside Sweden	0.8	0.0	1.2	1.1
*Time since graduation (years)*				
Mean (standard deviation)	1.8 (1.4)	1.8 (1.4)	2.0 (1.4)	2.7 (1.1)
*Occupation (%)*				
Employed	80.0	78.6	86.5	74.5
Student	12.0	15.7	3.8	8.5
Parental leave	5.6	2.9	9.6	17.0
Other *	2.4	2.9	0.0	0.0
*Workplace (%)*				
Community pharmacy	67.5	81.2	49.1	76.3
Hospital pharmacy	3.3	5.8	1.8	4.3
County council **	9.8	4.3	16.4	6.5
Pharmaceutical company	9.8	1.4	20.0	3.2
Other ***	9.6	7.3	12.7	9.7
*Employment category (%)*				
Employee	92.7	91.2	92.6	89.2
Manager	7.3	7.4	7.4	10.8
*Type of employment (%)*				
Permanent	80.0	77.9	85.4	89.2
Other	20.0	22.1	14.6	10.8
*Time until the first job after graduation (%)*				
Before completing education	80.6	80.9	80.0	86.2
Less than six months after	16.1	14.7	18.2	10.7
More than six months after	3.2	4.4	1.8	1.1
*Personal monthly income (SEK before tax) (%)*				
Less than 25,000	4.1	7.5	0.0	4.3
25,001–30,000	2.5	1.5	3.6	27.2
30,001–35,000	27.9	38.8	14.5	44.6
35,001–40,000	35.2	38.8	30.9	10.9
40,001–45,000	8.0	6.0	34.5	9.8
45,001 or more	11.5	7.5	16.4	3.3

* Other include unemployed and sick leave. ** County councils are responsible for the public healthcare systems in Sweden. *** Other include drug product manufacturing, dose dispensing pharmacy, university, and municipality. SEK = Swedish krona.

**Table 2:** Responses to questions regarding job satisfaction among pharmacy graduates in the two groups (graduation years 2015–2018 and 2019–2023).

Question	Proportion of Graduates (%) Responding “Most of the Time” or “All of the Time” to the Questions		
	2015–2018	2019–2023	*p*-Value
All things considered, how often are you satisfied with your job?	91.4 (85/93)	76.4 (94/123)	0.004
How often do you think the idea of spending the remainder of your working life in a job like your current one is depressing?	13.0 (12/92)	22.0 (27/123)	0.094
How often do you leave work with a “bad” feeling, a feeling that you are doing something you do not enjoy?	4.3 (4/93)	12.2 (12/123)	0.043
How often do you get so wrapped up (interested) in your work that you lose track of time?	39.1 (36/92)	36.6 (45/123)	0.703

**Table 3:** Logistic regression including various questions regarding job satisfaction among pharmacists who graduated between 2019 and 2023.

	People Satisfied with Their Job	People Not Satisfied with Their Job	Simple OR (95% CI)	Multiple OR (95% CI)
Cases, *n* (%)	94	29		
Women, *n* (%)	84/93 (90.3)	23/29 (79.3)	2.435 (0.785–7.547)	2.391 (0.628–9.107)
Age Mean ± SD	33.3 ± 8.5	36.7 ± 9.8	1.042 (0.996–1.090)	1.024 (0.971–1.080)
*Employment category*				
Manager, *n* (%)	6/93 (6.5)	3/29 (10.3)	0.598 (0.140–2.557)	
Employee, *n* (%)	87/93 (93.5)	26/29 (89.7)	Ref	
*Workplace*				
Community pharmacy, *n* (%)	58/94 (61.7)	25/29 (86.2)	0.258 (0.083–0.802)	0.241 (0.054–1.072)
Other, *n* (%)	36/94 (38.3)	4/29 (13.8)	Ref	Ref
*Personal monthly income (SEK before tax)*				
≥30,001 *n* (%)	85/91 (93.4)	27 (93.1)	1.049 (0.200–5.507)	
≤30,000, *n* (%)	6/91 (6.6)	2 (6.9)	Ref	
*Time since graduation (years)*	1.8 ± 1.5	1.7 ± 1.3	0.936 (0.701–1.250)	
*Access to CPD*				
Good, *n* (%)	76 (80.9)	9 (32.1)	8.914 (3.465–22.932)	5.360 (1.896–15.156)
Limited, *n* (%)	18 (19.1)	19 (67.9)	Ref	Ref
*The knowledge and skills acquired during the education are useful in my current job*				
Agree, *n* (%)	79 (84.0)	17 (58.6)	3.718 (1.478–9.351)	3.983 (1.255–12.642)
Disagree, *n* (%)	15 (16.0)	12 (41.4)	Ref	Ref

CI = Confidence Interval, CPD = continuing professional development, OR = Odds Ratio, Ref = Reference, SD = Standard Deviation, SEK = Swedish krona. The multiple regression includes significant variables in the simple regression (*p* < 0.05) as independent variables; opportunities for CPD, the knowledge and skills gained during the education are beneficial in my current job, and working in community pharmacy. Gender and age were also included in the analysis.

## Data Availability

The original contributions presented in this study are included in the article. Further inquiries can be directed to the corresponding author.

## References

[1] Westerlund T., Marklund B. (2020). Community pharmacy and primary health care in Sweden—At a crossroads. Pharm. Pract..

[2] Westerlund T., Söderlund L.Å. (2023). Role of community pharmacy and pharmacists in self care in Sweden. Explor. Res. Clin. Soc. Pharm..

[3] Wisell K., Winblad U., Sporrong S.K. (2016). Stakeholders’ expectations and perceived effects of the pharmacy ownership liberalization reform in Sweden: A qualitative interview study. BMC Health Serv. Res..

[4] (2023). Swedish Pharmacy Association. Sveriges Apoteksförening Sector Report 2023. https://sverigesapoteksforening.se/wp-content/uploads/2023/06/eng-juni-SVAP0011_Apoteksforeningen_Bran-schrapport_2023_ENG_20230622.pdf.

[5] Radwan R.M., Bentley J.P., Patterson J.A., Dixon D.L., Salgado T.M. (2022). Predictors of job satisfaction among pharmacists: A regional workforce survey. Explor. Res. Clin. Soc. Pharm..

[6] McCann L., Hughes C., Adair C., Cardwell C. (2009). Assessing job satisfaction and stress among pharmacists in Northern Ireland. Pharm. World Sci..

[7] Iqbal M.S., Al-Saikhan F.I., Ahmed N.J., Iqbal M.Z. (2020). Predictors of Job and Workplace Satisfaction among Community Pharmacists. J. Pharm. Res. Int..

[8] Manan M.M., Azmi Y., Lim Z., Neoh C.F., Khan T.M., Ming L.C. (2015). Predictors of job satisfaction amongst pharmacists in Malaysian public hospitals and healthcare clinics. J. Pharm. Prac. Rec..

[9] Gustafsson M., Mattsson S., Wallman A., Gallego G. (2018). Pharmacists’ satisfaction with their work: Analysis of an alumni survey. Res. Soc. Adm. Pharm..

[10] Mattsson S., Gustafsson M. (2020). Job satisfaction among Swedish Pharmacists. Pharmacy.

[11] Bonenberger M., Aikins M., Akweongo P., Wyss K. (2014). The effects of health worker motivation and job satisfaction on turnover intention in Ghana: A cross-sectional study. Hum. Resour. Health.

[12] Al-Muallem N., Al-Surimi K.M. (2019). Job satisfaction, work commitment and intention to leave among pharmacists: A cross-sectional study. BMJ Open.

[13] Bond C.A., Raehl C.L. (2001). Pharmacists’ assessment of dispensing errors: Risk factors, practice sites, professional functions, and satisfaction. Pharmacotherapy.

[14] Mott D.A., Doucette W.R., Gaither C.A., Pedersen C.A., Schommer J.C. (2004). Pharmacists’ Attitudes Toward Worklife: Results From a National Survey of Pharmacists. J. Am. Pharm. Assoc..

[15] Saari L.M., Judge T.A. (2004). Employee attitudes and job satisfaction. Hum. Resour. Manag..

[16] Berassa M.S., Chiro T.A., Fanta S. (2021). Assessment of job satisfaction among pharmacy professionals. J. Pharm. Policy Pract..

[17] Al-Omar H.A., Arafah A.M., Barakat J.M., Almutairi R.D., Khurshid F., Alsultan M.S. (2019). The impact of perceived organizational support and resilience on pharmacists’ engagement in their stressful and competitive workplaces in Saudi Arabia. Saudi Pharma J..

[18] Alshahrani S.M., Ishaqui A.A., Alavudeen S.S. (2025). Job satisfaction and its correlation with pharmacists’ performance and patient trust. Front. Med..

[19] Wang Y. (2024). Factors Affecting Employees’ Job Satisfaction: Organizational and Individual Levels. SHS Web Conf..

[20] Barakat M., Sallam M. (2025). Pharmacy workforce: A systematic review of key drivers of pharmacists’ satisfaction and retention. J. Pharm. Policy Pract..

[21] Swedish Pharmacists Association (2017). Rapport-Arbetsmiljöenkät för Farmaceuter på Apotek. https://mb.cision.com/Public/262/2189424/811c95355baa4a9d.pdf.

[22] Terry D., Phan H., Peck B., Hills D., Kirschbaum M., Bishop J., Obamiro K., Hoang H., Nguyen H., Baker E. (2021). Factors contributing to the recruitment and retention of rural pharmacist workforce: A systematic review. BMC Health Serv. Res..

[23] Ljungberg Persson C., Nordén Hägg A., Södergård B. (2025). Measuring the patient safety climate in community pharmacies: An updated national survey. BMJ Open.

[24] Statistics Sweden. https://www.statistikdatabasen.scb.se.

[25] National Board of Health and Welfare. https://www.socialstyrelsen.se/statistik-och-data/statistik/statistikamnen/halso-och-sjukvardspersonal/.

[26] Seston E., Hassell K., Ferguson J., Hann M. (2009). Exploring the relationship between pharmacists’ job satisfaction, intention to quit the profession, and actual quitting. Res. Soc. Adm. Pharm..

[27] Murawski M.M., Payakachat N., Koh-Knox C. (2008). Factors affecting job and career satisfaction among community pharmacists: A structural equation modeling approach. J. Am. Pharm. Assoc..

[28] Hardigan P., Carvajal M. (2007). Job Satisfaction Among Practicing Pharmacists: A Rasch Analysis. Internet J. Allied Health Sci. Pract..

[29] Carvajal M.J., Popovici I. (2018). Gender, age, and pharmacists’ job satisfaction. Pharm. Prac..

[30] Statistics on Licensed Health Care Personnel 2018 and Workforce Status 2017. https://www.socialstyrelsen.se/globalassets/sharepoint-dokument/artikelkatalog/statistik/2019-9-6312.pdf.

[31] Students and Graduates at First and Second Cycle Studies. Statistics on Number of Registered Students, Graduates and Gender. https://www.scb.se/contentassets/a67649a5a31949289650acfe4b53c1ef/uf0205_2023l24_sm_uf20sm2501.pdf.

[32] Krol E.S., Dobson R.T., Adesina K. (2019). Association between Prerequisites and Academic Success at a Canadian University’s Pharmacy Program. Am. J. Pharm. Educ..

[33] Taylor J.N., Nguyen N.T., Taylor D.A. (2018). The Pharmacy Student Population: Applications Received 2016-17, Degrees Conferred 2016-17, Fall 2017 Enrollments. Am. J. Pharm. Educ..

[34] Mattsson S., Gustafsson M., Svahn S., Norberg H., Gallego G. (2018). Who enrols and graduates from web-based pharmacy education-Experiences from Northern Sweden. Curr. Pharm. Teach. Learn..

[35] Abdullahi A.K., Mosanya A.U., Bello N., Musa M.K. (2023). Evaluation of job satisfaction among pharmacists working in public health facilities. Expl. Res. Clin. Soc. Pharm..

[36] Lam S.J., Lynd L.D., Marra C.A. (2023). Pharmacists’ Satisfaction with Work and Working Conditions in New Zealand—An Updated Survey and a Comparison to Canada. Pharmacy.

[37] Swedish Pharmacists Association (2023). Arbetsmiljöenkät. https://www.sverigesfarmaceuter.se/globalassets/2-dokument/2-rad-och-stod/arbetsmiljo/resultat-fran-var-arbetsmiljoenkat/arbetsmiljoenkat-sammanstallning-med-tabeller-2023.pdf.

[38] Aldaiji L.A., Al-Jedai A., Alamri A., Alshehri A.M., Alqazlan N., Almogbel Y. (2022). Effect of Occupational Stress on Pharmacists’ Job Satisfaction in Saudi Arabia. Healthcare.

[39] Jacobs S., Hassell K., Ashcroft D., Johnson S., O’Connor E. (2014). Workplace stress in community pharmacies in England: Associations with individual, organizational and job characteristics. J Health Serv. Res. Policy.

[40] Owens C.T., Baergen R. (2021). Pharmacy Practice in High- Volume Community Settings: Barriers and Ethical Responsibilities. Pharmacy.

[41] Lynch M., O’Leary A.C. (2023). Understanding the factors influencing community pharmacist retention—A qualitative study. Explor. Res. Clin. Soc. Pharm..

[42] Jairoun A.A., Al-Hemyari S.S., Jaber A.A.S., Shahwan M., El-Dahiyat F., Karuniawati H., Jairoun M., Alorfi N.M. (2022). Over-the-counter counseling in community pharmacies and job satisfaction among pharmacy professionals: A reflection of current scenario and possible solutions. Pharm. Pract..

[43] Johnston K., O’Reilly C.L., Scholz B., Mitchell I. (2022). The experiences of pharmacists during the global COVID-19 pandemic: A thematic analysis using the jobs demands-resources framework. Res. Soc. Adm. Pharm..

[44] Padiyara R.S., Komperda K.E. (2010). Effect of postgraduate training on job and career satisfaction among health-system pharmacists. Am. J. Health Syst. Pharm..

[45] Ibrahim I.R., Ibrahim M.I., Majeed I.A., Alkhafaje Z. (2021). Assessment of job satisfaction among community pharmacists in Baghdad, Iraq: A cross-sectional study. Pharm. Pract..

[46] Carvajal M.J., Popovici I., Hardigan P.C. (2018). Gender differences in the measurement of pharmacists’ job satisfaction. Hum. Resour. Health.

[47] Carvajal M., Popovici I., Hardigan P. (2019). Gender and Age Variations in Pharmacists’ Job Satisfaction in the United States. Pharmacy.

[48] Stewart J.E., Smith S.N. (1987). Work expectations and organizational attachment of hospital pharmacists. Am. J. Hosp. Pharm..

[49] Hardigan P.C., Carvajal M.J. (2008). Application of Rasch rating scale model to analysis of job satisfaction among practicing pharmacists. J. Am. Pharm. Assoc..

[50] Ayele Y., Hawulte B., Feto T., Basker G.V., Bacha Y.D. (2020). Job satisfaction among pharmacy professionals working in public hospitals and its associated factors, eastern Ethiopia. J. Pharm. Policy Pract..

